# Girls in early childhood increase food returns of nursing women during subsistence activities of the BaYaka in the Republic of Congo

**DOI:** 10.1098/rspb.2022.1407

**Published:** 2022-11-30

**Authors:** Haneul Jang, Karline R. L. Janmaat, Vidrige Kandza, Adam H. Boyette

**Affiliations:** ^1^ Department of Human Behavior, Ecology and Culture, Max Planck Institute for Evolutionary Anthropology, 04103 Leipzig, Germany; ^2^ Department of Evolutionary and Population Biology, Institute of Biodiversity and Ecosystem Dynamics, Faculty of Science, University of Amsterdam, 94248 Amsterdam, The Netherlands; ^3^ Department of Cognitive Psychology, Faculty of Social and Behavioral Sciences, Leiden University, 2333 Leiden, The Netherlands

**Keywords:** allomaternal care, cooperative subsistence activities, child helpers, age-graded cooperation, pooled energy model

## Abstract

Nursing mothers face an energetic trade-off between infant care and work. Under pooled energy budgets, this trade-off can be reduced by assistance in food acquisition and infant care tasks from non-maternal carers. Across cultures, children also often provide infant care. Yet the question of who helps nursing mothers during foraging has been understudied, especially the role of children. Using focal follow data from 140 subsistence expeditions by BaYaka women in the Republic of Congo, we investigated how potential support from carers increased mothers' foraging productivity. We found that the number of girls in early childhood (ages 4–7 years) in subsistence groups increased food returns of nursing women with infants (kcal collected per minute). This effect was stronger than that of other adult women, and older girls in middle childhood (ages 8–13 years) and adolescence (ages 14–19 years). Child helpers were not necessarily genetically related to nursing women. Our results suggest that it is young girls who provide infant care while nursing mothers are acquiring food—by holding, monitoring and playing with infants—and, thus, that they also contribute to the energy pool of the community during women's subsistence activities. Our study highlights the critical role of children as carers from early childhood.

## 1. Introduction

Compared to other great apes, humans have a life history characterized by relatively short interbirth intervals and high fertility, and thus a unique pattern of stacking highly dependent and costly offspring [1–4]. To simultaneously meet the demands of multiple offspring as well as the energetic cost of nursing, human mothers need the assistance of others [3,5]. Cooperative childrearing—defined as receiving childcare assistance from non-parental members of a group—increases maternal fertility [6–9] as well as child health and survivorship [9–13] by providing mothers with direct childcare [2,14–17] and/or food [3,6,14,18–22]. Human cooperation in childcare enables women to successfully rear multiple offspring and, thus, is essential in understanding the demographic success of humans in evolutionary history [3].

Alongside this, cooperation in childrearing enables mothers to invest more time and energy in other labour activities such as food acquisition [14,15]. Across cultures, mothers with nursing children face choices about the trade-off between childcare and work. In hunter–gatherer societies, nursing mothers either reduce foraging time for intensive infant care or spend time foraging but risk harm to infants, by either leaving them at camps away from their mother's care or by taking them on foraging expeditions [14,23–25]. Under a pooled energy model [24], allomaternal support may mitigate this trade-off, by allowing intensive childcare and foraging not to be mutually exclusive. Forager mothers with nursing children routinely receive assistance from allomaternal carers. When mothers remain at home with infants, allomaternal carers provide provisions for mothers and infants [10,26,27]. When mothers leave infants at camp and go foraging, carers at camp provide childcare and enable mothers to invest more time and energy in food acquisition [17].

Nursing mothers receive allomaternal support also when they take infants on foraging expeditions. In addition to the risk of harming infants by taking them on long-distance expeditions, carrying infants during such expeditions is one of the greatest drains on maternal energy [28]. Moreover, looking after infants during foraging activities may reduce the time and effort that mothers can put towards work. To reduce the burden of childcare on nursing mothers during foraging, accompanying individuals in foraging groups can provide direct childcare while nursing mothers are foraging, or take on food acquisition duties while mothers are looking after their infants (e.g. breastfeeding). Through this allomaternal support during foraging, group members may contribute to the pooled energy budgets of the subsistence group, for example, by increasing the collected food of the group [24]. Yet, the question of who supports nursing mothers during out-of-camp foraging activities is largely understudied, as is the impact of allomaternal support during foraging on the trade-off between childcare and food acquisition for mothers.

Hunter–gatherer women typically acquire food in variable-sized groups with a mix of adults and children [29], in which group members can play a role as potential carers during foraging activities. Across cultures, the role of kin in cooperative childrearing has been emphasized [1,10,11, 17,21,25,30]. To reduce the burden of childcare on nursing mothers, grandmothers and siblings look after a child and fathers contribute to provisions [3]. In particular, recent studies highlighted that older children are important carers to their young siblings by monitoring and holding infants, and by playing in children-only groups [6,9,14,19,31–33]. As forager children join their mothers on subsistence expeditions from early ages [34], they may also provide assistance in childcare when mothers engage in food acquisition [19]. However, hunter–gatherer children have not yet been demonstrated to participate much in the direct caring of young siblings compared to those in food-producing societies [1,35]. As most research has focused on how children provide childcare in camp settings, without considering the importance of children's support during labour activities, it is essential to investigate children's roles as carers during their mothers' foraging expeditions. We expect that this will allow us to understand the full picture of cooperative childrearing in humans from the perspective of a pooled energy model.

Here, we used focal follow data from 140 daily subsistence expeditions by four BaYaka nursing mothers in the Republic of the Congo. The BaYaka are a group of forest foragers, living in fluid communities across the northwestern Congo Basin. The BaYaka have been integrated into a mixed subsistence economy while practicing a wide range of subsistence activities from hunting and gathering to fishing, farming and trading [36]. Among the BaYaka, food is shared extensively. Childcare is also shared [35]; infants can be cared for by up to 20 carers each day [25]. At camp, BaYaka allomaternal carers range from fathers and grandmothers, who offer the most direct care to infants, to multiple individuals varying in age, sex and relatedness, including siblings, unrelated adults, and children [21]. To expand our knowledge of BaYaka allomaternal support in out-of-camp settings, we analysed data on subsistence group composition, food collection duration, and the weight of collected food at the end of subsistence expeditions for gathering forest plant foods, crop cultivating, and fishing. Given the literature on the trade-off between work and childcare for nursing mothers [3,14,37], we predicted that the work efficiency of BaYaka nursing mothers, defined as food returns per collection duration (kcal min^−1^) in this study, would increase with the number of potential carers in subsistence groups, particularly when mothers take nursing infants or toddlers (hereafter, *‘nursing children’*) on subsistence expeditions. We also included children as potential carers and further investigated how the effects of allomaternal support changed according to the kin status, gender and age class of the potential carers.

## 2. Results

We observed 272 out-of-camp expeditions for subsistence activities by BaYaka women over 230 days (1144 observation hours in total). Our sample of 272 subsistence expeditions reflects a history of daily decisions made by five BaYaka women. These five focal women represent 31.25% of the women usually residing in the camp where we collected data (a total of 16 women in the residential camp). Across the 272 expeditions, the expedition duration and travel distance of the five focal women were 4.20 h (s.d. = 2.55, range: 0.07–11.82 h) and 3.90 km (s.d. = 2.97, range: 0.11–13.70 km) on average, respectively. The five women participated in subsistence activities with two other adults and four children on average, with a range from zero to 11 adults and from zero to 14 children (table 1). Women collected 1273.34 kcal per daily expedition on average (s.d. = 2848.99, range: 2.78–18 023.64 kcal expedition^−1^). We followed 199 expeditions by four women who had nursing children at the time of observation and 73 expeditions by one post-reproductive woman whose youngest child was 6 years old and who already had grandchildren (electronic supplementary material, table S1). To investigate the effects of the presence of a woman's nursing child during out-of-camp expeditions on her food returns per collection duration, we excluded data from one post-reproductive woman. The nursing mothers took their dependent children on 109 of 199 expeditions in total: 56.13% on average (s.d. = 26.59, range: 34.38–77.27%), which varied depending on the age of the nursing children (electronic supplementary material, table S1). The younger the nursing children were, the higher the chance that mothers would take them on expeditions for subsistence activities (electronic supplementary material, table S1). BaYaka nursing children were carried by allomaternal carers for 40% of the total travel and work time during subsistence expeditions on average (s.d. = 5.05, range: 32.41–48.41%). We were able to weigh collected food items from 140 of the 199 expeditions by four nursing mothers before the food was distributed or processed.

**Table 1.** Summary of composition of four BaYaka women's groups on 140 subsistence expeditions.

	mean	s.d.	range (min–max)
group size	7.88	4.31	1–20
*n* adults including a focal woman	2.93	1.75	1–11
*n* kin adults (0 < *r* ≤ 0.5)	0.69	1.05	0–6
*n* non-kin adults (*r* = 0)	1.18	1.33	0–7
*n* kin children (0 < *r* ≤ 0.5)	1.55	2.12	0–11
*n* non-kin children (*r* = 0)	1.05	1.61	0–10
*n* women	2.82	1.42	0–7
*n* men	0.11	0.38	0–4
*n* girls	2.20	1.94	0–9
*n* boys	1.15	1.23	0–6
*n* adolescents (13 < *a* ≤ 19)	0.43	0.63	0–4
*n* independent children (4 ≤ *a* ≤ 13)	4.52	3.12	0–14
*n* nursing children (<4)	1.17	1.06	0–4
*n* girls in early childhood (4 ≤ *a* ≤ 7)	1.30	1.16	0–6
*n* girls in middle childhood (7 < *a* ≤ 13)	0.90	1.03	0–5
*n* girls in adolescence (13 < *a* ≤ 19)	0.35	0.58	0–4
*n* households	4.34	2.01	1–10

**Figure 1.**
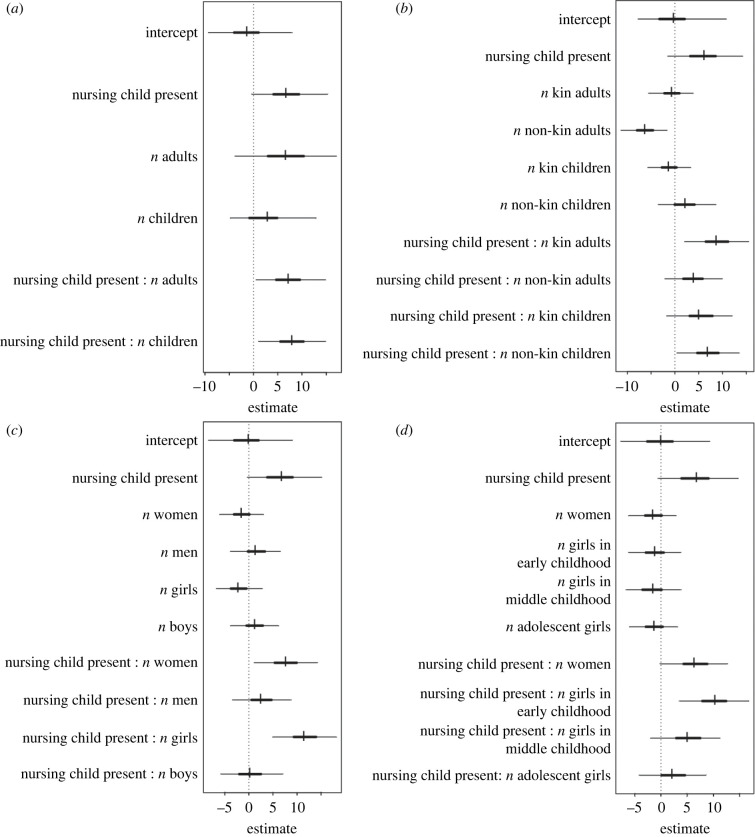
Estimated changes in focal women's food returns per collection duration (kcal min^−1^) with 80% and 95% credibility intervals, depending on the number of potential carers in groups: (*a*) adults and children, (*b*) kin status, (*c*) gender, and (*d*) age classes of females. The numbers on the *x*-axis are the standardized values of kcal collected per minute.

To investigate who contributes to the foraging productivity of nursing mothers (measured in kcal min^−1^) during 140 subsistence expeditions, we used Bayesian-estimated posterior samples, which optimize inferences for small sample sizes. First, we tested whether the number of adults (≥20 years) and children (from 4 to 19 years) who accompanied them on the subsistence expeditions affected the food returns of nursing mothers, in interaction with the presence of their nursing children (≤3 years) (model 1 in table 2). We found positive effects with the number of adults as well as the number of children on the food returns of nursing mothers, especially when the mothers took their nursing children on subsistence expeditions (model 1 in the electronic supplementary material, table S2, figure 1*a*). Second, we examined whether the kinship relationship between the mother and other subsistence group members affected her food returns (model 2 in table 2). We found that when nursing mothers went on subsistence expeditions with a greater number of kin adults (0 < *r* ≤ 0.5), the mothers had higher food returns, in the presence of nursing children in subsistence groups. This effect was also found when the mothers went on expeditions with non-kin children (*r* = 0) (model 2 in the electronic supplementary material, table S2; figure 1*b*). Third, we examined whether the gender of subsistence group members affected the food returns of nursing mothers (model 3 in table 2). We found positive effects with women and girls, but not with men or boys (model 3 in the electronic supplementary material, table S2; figure 1*c*). Fourth, we examined whether the age class of females in subsistence groups affected the food returns of nursing mothers (model 4 in table 2). We found that the number of girls in early childhood (from 4 to 7 years) on an expedition predicted increased food returns by the nursing mothers, especially when the mothers took their nursing children on subsistence expeditions. This effect was stronger than that of adult women (model 4 in the electronic supplementary material, table S2; figure 1*d*). However, we did not find any considerable effects with girls in middle childhood (from 7 to 13 years) nor in adolescence (from 13 to 19 years) (model 4 in the electronic supplementary material, table S2; figure 1*d*). The focal women, the expeditions, food types collected, or locations did not have any considerably different effects on food returns (electronic supplementary material, figure S1).

**Table 2.** Model structure overview.

	response variable	fixed effects	random effects
model 1	food returns per collection duration (kcal min^−1^)	nursing child present (yes/no) * *n* adults in a group	women identity (ID) expedition ID food type location type
		nursing child present (yes/no) * *n* children in a group	
model 2		nursing child present * *n* kin adults (0 < *r* ≤ 0.5)	
		nursing child present * *n* non-kin adults (*r* = 0)	
		nursing child present * *n* kin children (0 < *r* ≤ 0.5)	
		nursing child present * *n* non-kin children (*r* = 0)	
model 3		nursing child present * *n* women	
		nursing child present * *n* men	
		nursing child present * *n* girls	
		nursing child present * *n* boys	
model 4		nursing child present * *n* early childhood girls (4 ≤ a ≤ 7)	
		nursing child present * *n* middle childhood girls (7 < *a* ≤ 13)	
		nursing child present * *n* adolescent girls (13 < *a* ≤ 19)	
		nursing child present * *n* adult women (≥20)	

*n*_total data points_ = 223, *n*_focal women_ = 4, *n*_expeditions_ = 140, *n*_food types_ = 8, *n*_location types_ = 3.

## 3. Discussion

**Figure 2.**
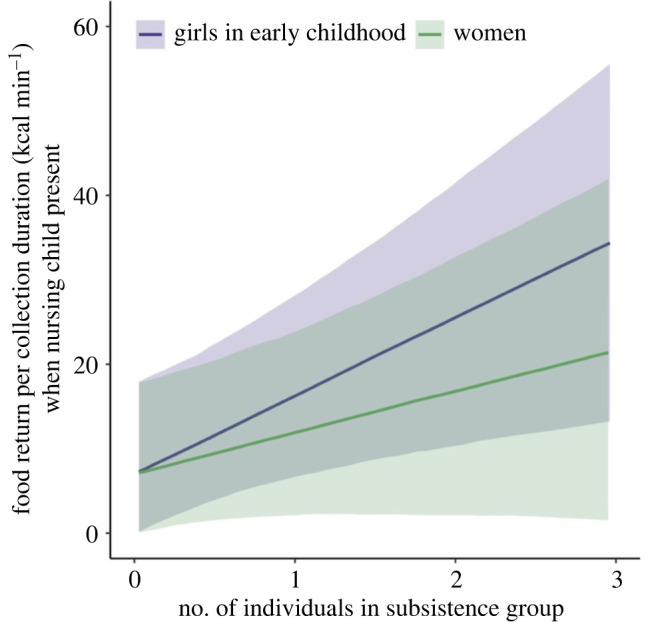
Predicted changes in food returns per collection duration (kcal min^−1^) depending upon the number of girls in early childhood (purple line) and other adult women (green line) in the nursing women's subsistence groups when nursing children were present in the groups. (Online version in colour.)

When mothers took their nursing children on subsistence expeditions, allomaternal support from accompanying women and girls increased the food returns per collection duration of the mothers. Crucially, girls in early childhood—but not in middle childhood or adolescence—had an even stronger effect on the nursing women's food returns than adult women (figures 1*d* and 2). Moreover, unlike in adult helpers where kinship played an important role, children seemed to be helpful to nursing women regardless of their relationship to the women (figure 1*b*). Our results highlight the critical role of children as carers from early childhood.

Adult women who accompany mothers in subsistence groups can provide direct childcare by carrying a child when mothers are walking or working. Unlike adult women, however, we rarely observed that young girls in early childhood carried nursing children while walking in the forest, probably because carrying an infant requires considerable energy [28]. In addition, adult women might have increased the nursing mothers' food returns by undertaking foraging duties when the mothers were attending to children, as BaYaka women commonly put collected food items in shared baskets. It is unlikely, however, that young girls collected food items as efficiently as adult women. We observed that BaYaka children often practiced digging wild yams or cracking nuts, but the amount that they collected would hardly match that of the women enough to offset the foraging cost of nursing women, as gathering requires a high level of skill and strength [38–40]. Instead, girls may reduce the burden of childcare for nursing women by holding, monitoring and playing with nursing children while their mothers work, as they were observed to do in camp ([6,13,21,26,32,33]; but see [14]). We indeed observed that young BaYaka girls stayed close to women and held infants or played with toddlers while women were digging wild yams or scooping water for bail fishing (electronic supplementary material, figure S2).

Unexpectedly, the contribution of adult women in allomaternal childcare was lower compared to that of young girls (figure 2). This is likely because adult women in subsistence groups may engage more in their own subsistence activities than in allomaternal childcare. We observed that even grandmothers engaged actively in their own food acquisition behaviours, when they went on subsistence expeditions with their daughters with nursing children. Yet, those adult women may still reduce the energetic burden on nursing women and contribute to the energy pool, by sharing collected or cooked food later at camp [24]. Alongside this, we did not find any considerable influences of girls in middle childhood and adolescence on the nursing mothers’ food returns (figure 1*d*). By middle childhood, girls have sufficient strength and skill to participate in the food acquisition and, thus, they are also likely to produce their own foraging outputs and share food at camp [41]. Our results suggest that girls in early childhood, however, are more likely to contribute to the energy pool of the community by providing childcare, and thereby increasing nursing women's food returns.

Our finding provides an insight into how each individual in the community, even girls in early childhood, can contribute to a community energy pool through an age-graded division of labour. This prospect emphasizes that individuals at all life stages are considered to be both energetic contributors to and receivers in a community, supporting the pooled energy budgets model [24,42]. Crucially, cooperation between nursing women and child carers during subsistence activities does not necessarily seem to occur among kin (figure 1*b*). This result suggests that children provide childcare not only for their siblings but also for other unrelated young children in the community. This could be because young girls are able to obtain additional food from unrelated women when they return to camp in return for childcare, or because young girls get opportunities while providing childcare to observe and learn from other women, not only from their mothers. We acknowledge that our data considered only the composition of subsistence groups and not the specific behaviours of carers. To confirm the extent of support with infant care by children during women's subsistence expeditions, detailed observational data focusing on children's caring behaviours are necessary. Moreover, observations on activities of girls in middle childhood and adolescence in women's subsistence groups are also necessary, to better understand how each individual contribute to a pooled energy budget depending on their skills and capacity. For future studies, we suggest investigating bidirectional transfers between nursing women (as teachers of foraging skills as well as receivers of childcare) and children (as social learners as well as carers) during subsistence activities, to understand the mutual benefits of mother–child cooperation associated with social learning and childcare.

One of main differences between human and nonhuman cooperative breeders is two classes of carers found only in human societies—grandmothers and older children [3]. Previous studies of BaYaka children—Aka and Bofi—indicate that children provide relatively little active care [35]. However, Aka children are actively taught how to care for infants [43]. As noted in Page *et al*. [31], passive care such as monitoring infants is a low-cost yet valuable type of childcare. Our study adds, to our knowledge, the first empirical evidence on the important role of children as allomaternal carers, by suggesting that infant care by young children may enable mothers to reduce their childcare burden during subsistence work and, therefore, increase food returns. This contribution of children as carers in out-of-camp settings, however, can vary depending on ecological environments and, thus, children's opportunities to join adults on foraging expeditions. To have a complete picture of human cooperative breeding, cross-cultural comparisons on how different ecology shapes variation in the level of children's contribution during out-of-camp foraging expeditions are necessary. Our findings highlight that the BaYaka's mixed-age groups during subsistence activities facilitate the flexible pooling of childcare labour, by involving carers from different age classes. This flexibility in allomaternal carers may have been an important key to a broad, co-evolutionary account of human life-history evolution [44].

## 4. Material and methods

### (a) The BaYaka

The BaYaka are a group of several populations of Congo Basin forest foragers who practice mixed-subsistence activities which involve hunting and gathering, fishing, cultivation, wage labour and trade. With some individual and group variation in the extent to which they engage in wild food procuring activities in forests or in cultivating crops, both BaYaka men and women actively participate in daily subsistence activities including gathering wild plants, fishing and hunting [36,45]. BaYaka men primarily collect honey and hunt with spears or guns, while women collect fruits, nuts, mushrooms and wild yams. BaYaka children often join adults during subsistence activities and also start foraging independently from the age of around 5 years [32,39,46]. Some BaYaka individuals or families are more mobile and spend more time in forests, whereas others are more sedentary and go foraging in the forest or work in crop fields while staying in villages and trading forest foods with villagers [36,47]. We conducted this study in one camp in the forest near a village along the Motaba River (the specific village name is not mentioned for privacy reasons, but the authors can provide more information when contacted). During our study period, camp composition fluctuated as some individuals came and left, consisting of 47 individuals on average (range: 20–79 individuals) including adults, children and infants from 10 households on average (range: 8–13 households).

### (b) Data collection

Data collection occurred over two field seasons from March to August in 2015 and 2016, respectively. We conducted genealogical interviews at the beginning of the data collection period. We had information on the exact birth year only for children who were born after 2014 [46,48]. To estimate the ages of older children, we used inter-birth intervals of 2 to 2.5 years. For adults, we conducted detailed interviews with BaYaka adults of variable ages and Bandongo villagers to estimate the ages of BaYaka adults by comparing them with the known ages of villagers at the end of the study period. We conducted focal follows of the daily subsistence expeditions of five BaYaka women (electronic supplementary material, table S1). We followed each focal woman's expeditions from the moment the focal woman left the camp until her return. We followed the same focal women twice for consecutive days in both 2015 and 2016. During a total of 230 observation days, focal women's expeditions were followed for a mean of 46 days (range: 40 to 53 days). We collected behavioural data in combination with a Garmin hand-held Global Positioning System (GPS; Garmin 62) and a voice recorder. We recorded the time when focal women began and finished foraging-related behaviours such as collecting nuts, digging wild yams, fishing or hunting. From these datasets, the collection duration of each food item was calculated. We recorded the presence or absence of the focal women's nursing infants for each expedition. During focal follows, we continuously recorded expedition group composition and identified each individual in the group. When focal women returned to camp at the end of expeditions, we measured the wet weight of the edible part of the collected items in the women's basket with a scale before the food was shared or cooked. We were able to weigh collected food items from 140 of the 199 expeditions by four women before the food was distributed or processed. We were unable to weigh food items that were cooked and consumed on-site in the forest during expeditions, such as a part of a fish, caterpillars or animals that were caught. From 140 expeditions, we were able to weigh 223 food items, categorized into animals (one species), fishes (multiple species), nuts (five species), wild yams (five species), caterpillars (four species), mushrooms (five species), leaves (three species) and crops (seven species), indicating that BaYaka women collected more than one food item per expedition on average (ranging from one to four food items). Species of collected food items were identified with the help of botanists from the Herbarium at the Institute de Recherche en Sciences Exactes et Naturelles (IRSEN) in Brazzaville in the Republic of the Congo.

### (c) Food returns per collection duration

To calculate food returns per collection duration, we used 140 expeditions by four nursing women on which we collected food returns data—including fishing, forest foraging, and crop cultivation in their own gardens or those of neighbouring Bandongo farmers, for whom the BaYaka often perform labour exchange for produce or other needs. Botanical identifications of food plants were performed at the Institut de Recherche en Sciences Exactes et Naturelles (IRSEN) Herbarium in Brazzaville. After identifying food item species, we calculated the energetic calories of each food item collected—by multiplying the total wet weight of the edible part of a food item by the reported value of energetic calories. We used the reported calorific values per the wet weight of the edible part of the known food species, from Kitanishi [47] as well as from the nutritional database of the United States Department of Agriculture (USDA) [49]. In the case that a food species was not listed in either Kitanishi [47] or the USDA database, we used the values provided for a known type in the same category (electronic supplementary material, table S3). We calculated food returns per collection duration by dividing the total energetic calories of a food item collected on an expedition by the total collection duration for that food item across the expedition in minutes (kcal min^–1^). Collection duration was defined as working time in minutes from searching to food acquisition, which varied depending on food types: the total duration of (i) searching and picking caterpillars, mushrooms and leaves, (ii) building dams, scooping water, searching and catching fishes, (iii) chasing, making holes on tree logs and killing porcupines, (iv) digging wild yams, (v) picking and cracking nuts, and (vi) digging and collecting crops, such as cassava, taro and maize. These six food types were included in the statistical models as random variables to account for possibly different effects of food items. A limitation of the study is that we measured the weight of food items only at the end of expeditions. Hence, we could not (i) calculate the efficiency of each foraging bout, (ii) include the food consumed in the forest, and (iii) separate the food collected by a focal woman and the food added by other individuals in subsistence groups.

### (d) Statistical analyses

We used Bayesian multilevel regression models in the Stan computational framework (http://mc-stan.org/), accessed with the function ‘brm’ of the brms package v. 2.16.3 [50] in R v. 4.1.0 [51]. The unit of analysis in the statistical models was food returns per collection duration for each food item collected by a focal woman during a subsistence expedition. We used energetic kilocalories of each food item collected per minute as a response variable. We fitted models with a Gaussian error distribution. As group composition fluctuated during an expedition, we calculated the mean number of potential carers who appeared in the focal woman's expedition group and used them as model variables. We log-transformed the number of adults and the number of children. All quantitative predictors were then *z*-transformed to a mean of zero and standard deviation of one before fitting the model [52]. We included random effects of focal woman identity, expedition identity, targeted food type and location type (table 2). The models included interpretable random slopes for the fixed effects within random intercepts [53,54]. The models were based on 223 measured datasets of the food returns per collection duration of four nursing women, with expedition identities, food types (animal/caterpillar/fish/leaves/mushroom/nuts/wild yams/garden crops) and activity locations (forest/river/garden). We used weakly informative normal priors to guard against overfitting [55]. We included two two-way interactive effects between the presence of the women's nursing children in expedition groups (yes/no) and (i) the mean number of adults and (ii) the mean number of older children in the expedition group (model 1 in table 2). We obtained posterior distributions of the effects of predictors from four independent Markov chain Monte Carlo chains each with 4000 warmup and 2000 sampling iterations. To investigate the effect of kin status in the second model, we included four two-way interactive effects between the presence of the women's nursing children in expedition groups and (i) the number of kin adults, (ii) the number of non-kin adults, (iii) the number of kin children, and (iv) the number of non-kin children, respectively (model 2 in table 2). Third, to examine gender effects, we ran a model with four two-way interactive effects between the presence of the women's nursing children in expedition groups and (i) the number of women, (ii) the number of men, (iii) the number of girls, and (iv) the number of boys, respectively (model 3 in table 2). After we found that the presence of girls had a noticeable effect, we ran a model to further investigate which age class(es) of girls helped nursing women, with four two-way interactive effects between the presence of the nursing children and (i) the number of adult women, (ii) the number of girls in early childhood, (iii) the number of girls in middle childhood, and (iv) the number of girls in adolescence, respectively (model 4 in table 2).

## Data Availability

Data are available from the Dryad Digital Repository: https://doi.org/10.5061/dryad.rfj6q57dh [56]. All data and code used in analysis is uploaded in the GitHub Repository. Data are also provided in the electronic supplementary material [57].
